# Evaluation of the Early Outcomes of Laser-Endoscopic Pilonidal Sinus Treatment Combination and Comparison With the Combination of Cautery-Phenol-Endoscopic Pilonidal Sinus Treatment

**DOI:** 10.7759/cureus.26948

**Published:** 2022-07-17

**Authors:** Mustafa Dönmez, Murat Uludag

**Affiliations:** 1 General Surgery, Ankara Yildirim Beyazit University, Ankara, TUR; 2 General Surgery, Ankara Bilkent City Hospital, Ankara, TUR; 3 General Surgery, 29 Mayis State Hospital, Ankara, TUR

**Keywords:** minimally invasive surgery, endoscopic pilonidal sinus treatment, phenol, laser, pilonidal sinus disease

## Abstract

Introduction

Although there are several methods used in the treatment of pilonidal sinus, research is still ongoing for the most effective method. Minimally invasive surgical methods, alone or in combination, are currently considered the closest treatment modalities to the ideal. The purpose of this study was to evaluate the early outcomes of laser-endoscopic pilonidal sinus treatment combination and compare it with the combination of phenol-cautery-endoscopic pilonidal sinus treatment.

Materials and methods

This is a retrospective study of 42 patients with pilonidal sinus disease treated between September 2020 and April 2022. A total of 26 participants in group one were treated with the laser-endoscopic pilonidal sinus treatment combination, and the remaining 16 in group two were treated with the cautery-phenol-endoscopic pilonidal sinus treatment combination. Both primary and recurrent patients over 16 years of age without active infection were included. In the postoperative period, each patient was followed up at the outpatient clinic. Perioperative and follow-up data were recorded.

Results

Patients were predominantly male. There was no significant difference between the two groups with regards to the time to return to daily life, pain-free walking, recovery time, and recurrence. However, in group one, the operation time was statistically shorter (p = 0.02), and the rate of sitting on the toilet without pain on the day of surgery was significantly higher (p = 0.029). In addition, none of the patients in this group needed painkillers and all returned to work earlier.

Conclusion

The combination of laser-endoscopic pilonidal sinus treatment is a feasible procedure with a 92.3% complete recovery rate according to the early results. However, studies with a larger sample size and longer follow-up period are required to confirm the validity of our results.

## Introduction

Pilonidal sinus disease (PSD), first described by Mayo in the 19th century, is an inflammatory pathological condition, mostly located in the sacrococcygeal region. Generally, PSD affects younger males. Its presentation can range from asymptomatic to chronic pain, discharge, and acute exacerbations with abscess formation [1-3]. Possible risk factors (besides being male) include obesity, sitting for long periods, having excessive body hair, and inadequate hygiene [4,5].

Since the description of the disease, many surgical methods have been applied for treatment such as excision and primary midline closure, excision and marsupialization, and excision and closure with various flap techniques. Although these applications for treatment have different advantages and disadvantages, an ideal treatment method has not yet been determined [2,4,5]. The aims of a preferred method of treatment are short hospital stays, low complication rates, minimal postoperative pain, low wound infection rates, decreased use of wound dressings, quick return to daily life and work, low cost, low recurrence rates, and acceptable cosmesis [6-10].

In recent years, minimally invasive techniques have emerged. One of these techniques included curetting the sinus cavity and then filling it with phenol or fibrin glue. Meinero and Milone introduced endoscopic pilonidal sinus treatment (EPSiT) and video-assisted ablation of pilonidal sinus (VAAPS) techniques, respectively [2,11,12]. More recently, laser applications have also been used for the treatment of PSD [5,13-17]. And nowadays, efforts are still ongoing to find the optimal treatment by combining the listed methods [5,18-20].

We aimed to evaluate the efficacy and feasibility of endoscopic pilonidal sinus surgery combined with laser therapy. Parameters such as operation time, length of hospital stay, time to sit on the toilet without pain, time to return to daily life and work, complications, and recurrence rates were evaluated. Also, we aimed to compare this method with another minimally invasive treatment technique to determine the differences between them. So, in the current study, we evaluated the short-term outcomes of laser application combined with EPSiT and, to the best of our knowledge, for the first time, compared this treatment with the combination of EPSiT and phenol-cautery utilization.

## Materials and methods

This is a retrospective cohort study of patients with PSD who were treated between September 2020 and April 2022. It was approved by Ankara City Hospital No. 1 Clinical Research Ethics Committee (approval number: E1-20-1151) and carried out in accordance with the Helsinki declaration. A total of 42 patients were included in the study and informed consent was obtained. Demographic, perioperative, and follow-up data were recorded.

Patients over 16 years old with primary or recurrent PSD were evaluated; however, the ones with chronic diseases that may affect wound healing and those with signs of acute infection were excluded.

The patients were evaluated in two groups according to the treatment methods applied. First, detailed information was given to the patients about both treatment methods, and then the doctor and the patient decided together which procedure to be applied. In the first group (n = 26), laser therapy was added to the EPSiT, and in the second group (n = 16), cautery-phenol application was added.

All patients were hospitalized on the day of surgery. No patient was given prophylactic antibiotics. In addition, none of the patients were prescribed antibiotics in the postoperative period. Considering the patient's request and the doctor's preference, local or spinal anesthesia was applied.

Complete wound healing was defined as the complete closure of the wound, without spontaneous or palpable discharge, pain, and infection. Delayed recovery was assessed as persistence of symptoms even two months after the operation. Recurrence was the reappearance of symptoms four months after complete healing.

The patients were called for follow-up on the first, seventh, and 30th postoperative days. In addition, those who had problems were re-examined and re-evaluated immediately. In months six and 12, patients were followed-up by phone calls and/or photographs of the surgical site.

Surgical technique

Following induction of anesthesia, in the prone position, sinus openings were identified. The lengths and connections of the tracts were investigated with a metal probe. Subsequently, the sinus opening was enlarged to allow the passage of the endoscope, and the cavities were evaluated. The hairs in the tracts and sinus cavity were removed, and then curettage was performed and cleaned. After these procedures, all tracts and sinus cavities were observed again with the endo camera. If there was residual hair, it was removed under direct vision and washed to make sure that they were completely cleaned. Glycine (1%) was used as a solution for washing and endo camera use.

**Figure 1:**
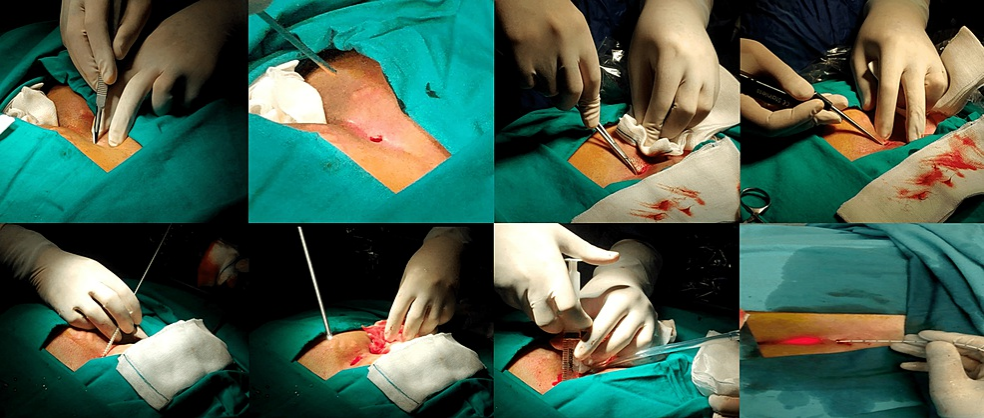
Stages of laser-endoscopic pilonidal sinus treatment combination

A radial laser probe with a wavelength of 1470 nm was placed on the cleaned tracts, and approximately 100-110 joules of energy was applied to 1 cm long areas at 10 watts. The laser probe was withdrawn 1 cm and applied throughout the entire tract (Figure 1). In patients treated with phenol, first, all the walls were cauterized under direct vision with a monopolar cautery placed through the endo camera, and then the entire tract and sinus cavity were filled with phenol.

Statistics

Statistical analysis was performed using Statistical Package for the Social Sciences version 20.0 (IBM Corp., Armonk, NY). Data were analyzed with Student's t-test, Mann-Whitney U test, and chi-square test as appropriate. Results were presented as frequency (n), percentage (%), mean ± standard deviation (SD), and median (minimal and maximal range). A p-value less than 0.05 was considered statistically significant.

## Results

A total of 42 patients were included in the study (Table 1). Of them, 26 were treated by adding laser application to EPSiT (Group 1), and 16 by adding cautery-phenol (Group 2). There were 39 (92.9%) males and three (7.1%) females. The mean age was 24.4 ± 6.8 years in the first group and 23.9 ± 6.2 in the second group, and no statistical difference was found (p = 0.794). Body mass index (BMI) was 27.8 ± 4.6 in the first group and 24.8 ± 2.8 in the second group, and the results were significant in favor of the first group (p = 0.021). There were 15 (57.7%) smokers in the first group and eight (50%) in the second one. Although the number of smokers was slightly higher in the first group, there was no significant difference in the results in favor of any group (p = 0.627).

**Table 1:** Characteristics of patients Lateral expanded: patients with only lateral orifices or both lateral and midline orifices.

Variables	Group 1 (n = 26)	Group 2 (n = 16)	P-value
Age (mean ± SD)	24.4 ± 6,8	23.9 ± 6.2	0.794
Gender (male/female, n)	23/3	16/0	N/A
BMI (mean ± SD)	27.8 ± 4.6	24.8 ± 2.8	0.021
Smoking (n, %)	15 (57.7%)	8 (50.0%)	0.627
Previous abscess drainage (n, %)	7 (26.9%)	4 (25.0%)	0.224
Primary/recurrence (n, %)	2 (7.7%)	2 (12.5%)	0.627
No. of sinus orifices (median, range)	2.2 (1-5)	1.9 (1-5)	0.323
No. of patients with ≥ 3 orifices (n, %)	9 (34.6%)	4 (25.0%)	0.513
Lateral expanded sinus orifices (n, %)	15 (57.7%)	6 (37.5%)	0.204
Local anesthesia (n, %)	4 (15.4%)	5 (31.3%)	0.224

The number of patients with previous abscess drainage was seven (26.9%) in the first group and four (25%) in the second group, and we could not find any statistical difference (p = 0.224). There were four relapsed cases in the study, two of them (7.7%) were in group one, and the remaining (12.5%) were in group two, and no significant difference was detected (p = 0.627). While the patients recovered successfully after the first group treatment, one patient in the second group relapsed again in the 11th month, but complete wound healing was achieved on the 10th day after the phenol application. The median number of sinus orifices was 2.1 (1-5). On the basis of groups, this number was 2.2 (1-5) in the first one and 1.9 (1-5) in the second, and there was no statistical difference (p = 0.323). We also evaluated sinus orifice localizations. The number of patients with midline orifice was 11 (42.3%) in group one and 10 (62.5%) in group two. Although the lateral orifice ratio was higher in the first group, there was no statistical difference (p = 0.204).

Local anesthesia was applied to four (15.4%) patients in the first group and five (31.3%) patients in the second group; the remaining cases were operated under spinal anesthesia. The median operative time was 25 (15-45) minutes and 40 (20-65) minutes, respectively, and this was statistically significant (p = 0.02; Table 2). Except for two patients from the second group, no one needed painkillers. All cases were day case procedures, and no statistical difference was found considering the mean of hospital stay (8.8 ± 5.5 hours and 7.5 ± 4.6 hours, respectively, p = 0.452).

**Table 2:** Postoperative and follow-up results * Day of surgery; ** postoperative first day.

Variables	Group 1	Group 2	P-value
Operation time (median, range, minute)	25 (15-45)	40 (20-65)	0.02
Hospital stay (mean ± SD, hour)	8.8 ± 5.5	7.5 ± 4.6	0.452
Pain-free sitting on the toilet (n, %)*	20 (76.9%)	7 (43.8%)	0.029
Time to start work (n, %)**	24 (92.3%)	12 (80.0%)	0.506
Complete healing time (mean ± SD, day)	17.3 ± 6.4	14.8 ± 4.3	0.17
Recurrence (n, %)	2 (7.7%)	1 (6.3%)	0.679

The median follow-up time was eight (3-19) months. A localized abscess developed on the 14th postoperative day in one patient in group one. It was drained under local anesthesia, no additional treatment was applied, and hospitalization was not required. In addition, a patient in the second group who underwent spinal anesthesia developed a headache, was hospitalized on the third postoperative day, and was discharged after two days of treatment. All patients were able to walk without pain on the day of surgery and returned to daily life on the first postoperative day. When the patients were evaluated in terms of time taken to sit on the toilet without pain, it was found that 20 (76.9%) of the patients in the first group and seven (43.8%) of the patients in the second group were able to do this on the day of surgery, and it was found to be statistically significant (p = 0.029). Considering the rate of starting to work on the first postoperative day, it was seen that the rate of the first group was higher than the second one (92.3% and 80%, respectively), but no statistical difference was detected (p = 0.506). The mean complete healing time was 17.3 ± 6.4 days in the first group and 14.8 ± 4.3 days in the second group, which indicates no notable difference (p = 0.17). Recurrence was observed in three patients in total. Two of them (7.7%) were from the first group and one (6.3%) from the second group, and we could not indicate any statistical difference. Relapse occurred in the first case in the fifth month, phenol was applied two times at one-week intervals, and healing was completely achieved in three weeks. The other case developed recurrence in the sixth month but was lost to follow-up. In the last case, recurrence developed in the 11th month, and complete recovery was achieved on the 10th day after phenol application.

## Discussion

PSD is a common surgical disease in men between the ages of 15 and 30 years. Although many surgical techniques have been tried, research is still ongoing for optimal treatment. Over time, minimally invasive surgical methods have gained popularity and successful results have been obtained [11,13,16,21,22]. Following this, combined minimally invasive techniques have been put into practice [19,20]. In the present study, we compared two combined minimally invasive techniques and also evaluated the early results of the combination of EPSiT and laser therapy. Male predominance and mean age were consistent with previous studies.

With reference to prior studies completed, in a study, the length of hospital stay of the patients was not mentioned but reported that the patients were discharged 30 minutes after the procedure was performed under local anesthesia [15]. In another study, the median hospital stay was reported as three hours, and the procedures were also performed under local anesthesia [14]. Bayhan et al. stated that patients who applied crystallized phenol were discharged after the procedure, and those who underwent the Limberg flap procedure were discharged after an average of 1.25 ± 0.4 days [10]. Kayaalp et al. reported that those who were administered local anesthesia had been discharged immediately after the procedure, while those who received general or regional anesthesia were discharged the following day after surgery [8]. Although the type of anesthesia and the order of surgery during the day are also important, one goal of minimally invasive surgery is to shorten the hospital stay. In our study, the duration of hospital stay was acceptable. Although there was no statistically significant difference, we attribute the slightly shorter hospital stay in the second group to the higher rate of spinal anesthesia in the first group.

In this study, it is clearly evident that the use of minimally invasive methods was successful in terms of shortening the operation time [6]. In former studies, it was defined that the operation time varied between 20 and 45 minutes in patients who underwent endoscopic pilonidal sinus surgery, and between six and 65 minutes for patients who underwent laser therapy [2,23]. Gulcu and Ozturk found the median operation time as 22 (16-38) minutes in their study in which these two methods were combined and called laser-assisted EPSiT (LEPSIT) [20]. With respect to the statistical analysis made, we found median operation time consistent with previous studies.

Minimally invasive pilonidal sinus treatment modalities were successfully performed with general anesthesia, regional anesthesia, or local anesthesia [1,10,11,13,15,19,20]. We also successfully completed the operations with either spinal or local anesthesia.

With minimally invasive surgery, it is aimed to achieve pain-free walking, pain-free sitting on the toilet, and returning to daily life and work as soon as possible. Milone et al. compared VAAPS and sinusectomy and did not find a significant difference in days off-work times (2.00 ± 1.30 days and 2.08 ± 1.24 days, respectively, p = 0.769) [3]. Milone et al., in another study on VAAPS, found the time to sit on the toilet without pain as 2 ± 1, the time to walk without pain as 1 ± 1, and the time off from work as 3 ± 3 days [12]. Calikoglu et al. compared phenol application and open healing procedures and reported that pain-free mobilization (0.8 ± 2.8 hours and 9.3 ± 10.0 hours, respectively, p < 0.001) and pain-free defecation (16.2 ± 12.6 hours, 22.5 ± 15.1 hours, respectively, p = 0.008) occurred significantly earlier in the phenol group [6]. Kayaalp and Aydin indicated that the mean time to return to work was 2.3 ± 3.8 days (range: 0-11.6 days) in their review study for phenol application [8]. Emile et al., on the other hand, indicated that the time to return to work period ranged from 1.6 to six days in their review study for EPSiT [2]. In a study on laser treatment, Georgiou stated that 53.3% of patients were able to return to work on the day of surgery. It could be inferred from his study that 25% of patients returned to work after three days or longer [15]. In another study, laser treatment and lay open procedure were compared and it was revealed that both pain-free walking (3.9 ± 1.1 days and 9.4 ± 1.4 days, respectively, p < 0.0001) and returning to work times (6.8 ± 1.6 days and 12.2 ± 2 days, respectively, p < 0.0001) were significantly shorter in laser treatment [17]. In their study combining EPSiT and phenol application, Gecim et al. determined that the mean time to return to work was 3.03 ± 2.95 days [19]. Gulcu and Ozturk compared EPSiT and LEPSIT methods in their recent study and reported that the aforementioned methods did not make a difference in the return time of patients to their daily activities (p = 0.242). Nonetheless, they found that those treated with the LEPSIT method returned to work in a significantly shorter time (three (1-11) days and two (1-6) days, respectively, p = 0.03572) [20]. In our study, all of the patients from both groups started to walk without pain on the day of surgery and could return to their daily life on the postoperative first day. Considering the previous studies, we can say that we have achieved successful results. At a later stage, we compared the two methods in terms of the pain-free sitting time on the toilet and concluded that the patients in the first group were able to achieve this significantly earlier and 76.9% could do this on the day of surgery. In addition, although there was no statistical difference, the rate of returning to work on the first postoperative day was found higher in the first group.

Likewise, smoking and obesity affect wound healing [24]. Moreover, there are studies evaluating the factors affecting the complete healing time and recurrence. For example, Bayhan et al. stated in their study that high BMI was associated with relapse [10]. Kaymakcioglu et al. reported that the number of sinus orifices was effective in the recurrence of the disease [21]. With similar justification, Guner et al. recommended more extensive surgical procedures, such as the Bascom cleft lift or rhomboid flap, instead of the pit-picking technique in patients with a high number of pits [25]. In another study, it was determined that the success of treatment decreased with the increase in the number of sinus openings. In the same study, it is also stated that the risk of failure in treatment was higher in patients with previous abscess drainage [9]. In their study, Meinero et al. reported that as the number of external openings increased, the recovery rate decreased. They found that the rate of failure in healing was 3.9% in those with a single external opening and it was 22.2% in those with three or more openings. In addition, they stated that the number of external openings was also effective on the healing time, and showed that those with one opening healed in an average of 25 days, and those with three or more openings healed in 33.9 days [26]. Meinero et al. further confirmed the negative effect of an increase in the number of external openings on wound healing in another study [4]. In a study comparing the phenol application and the open healing technique, it was shown for the phenol group that the recurrence rate was 15% in those with less than three openings, and 40% in those with three or more openings, and it was advised that surgery would be more appropriate in this group [6]. Additionally, Dessily et al. reported that the presence of secondary off midline orifices is important in patients with recurrence [16]. In our study, BMI was statistically higher in the first group. Although they were not statistically significant, the number of patients with previous abscess drainage, the number of sinus orifices, and the number of lateral orifices were higher in the first group. Despite this, the rate of complete recovery was determined as 92.3% with a single session application in the first group.

Phenol application has been used for a long time for the treatment of PSD, it is a simple and inexpensive method that can be performed with local anesthesia. Conversely, it has been reported in studies that it may cause skin burn, necrosis, cellulite, and abscess after application. It is important to note that repetitive applications are required to increase the success rate of phenol application, and the success rate with a single application is around 56%-86% [6-10,21,27]. Although laser application is a relatively new method for the treatment of PSD, it has been used safely for a long time in diseases such as perianal fistula, hemorrhoids, and varicose veins. Its advantages are as follows: it has a success rate of up to 97% in the first application, it can be used in every case regardless of the number and location of sinus orifices, it has a short learning curve, with an easy and repetitive application, and it can be performed as an outpatient procedure with local anesthesia. Another important advantage is the minimal effect on the surrounding tissue [13,15,16,23,28]. The techniques mentioned above are blind methods, for this reason, recently, researchers have started to apply a combined treatment method by adding video-assisted or EPSiT procedures. In this way, they reported that the sinus anatomy would be evaluated better, all the hairs could be cleaned, and it would be ensured that the entire epithelium was destroyed [5,13,16-20]. We performed the laser-EPSiT combination with the same goals and achieved successful results.

As a result, we evaluated the short-term results of EPSiT combined with laser application. In addition, we compared for the first time with another minimally invasive surgical treatment, the cautery-phenol-EPSiT combination. We think that our study meets our goals with its early results. But there are some limitations of our study such as the low number of samples and relatively short follow-up period. Nevertheless, we think that this situation does not prevent us from evaluating the early results of laser-EPSiT combination and obtaining beneficial results. Another shortcoming of our study is the cost evaluation. Although it is an accepted fact that laser therapy is costly, it is also stated that this situation can be tolerated by the short hospital stay, the absence of postoperative pain that results in no use of painkillers, limited workforce loss, and decreased postoperative care [13,16]. We are of the same opinion on this issue.

## Conclusions

We thought that the combination of laser-EPSiT fulfills the requirements of minimally invasive surgery due to short operation time, no need for postoperative painkillers, short hospital stay, returning to work in a short time, and low recurrence rate. In addition, the learning curve is short and it is an easily applicable treatment method. It is also a feasible technique that can be used for both primary and recurrent cases with local anesthesia. Although the equipment used is costly, we think that the advantages of mentioned combination can compensate for the drawback. It is justifiable to believe that it may be the first choice in the treatment of patients with pilonidal sinus. However, new studies with longer follow-up periods and a larger number of patients are needed.
